# Platelet P2Y _12_ Receptor Deletion or Pharmacological Inhibition does not Protect Mice from Sepsis or Septic Shock

**DOI:** 10.1055/s-0041-1733857

**Published:** 2021-08-24

**Authors:** Yannick Rabouel, Stéphanie Magnenat, Xavier Delabranche, Christian Gachet, Beatrice Hechler

**Affiliations:** 1 Université de Strasbourg, INSERM, Etablissement Français du Sang (EFS)-Grand Est, BPPS UMR_S 1255, Fédération de Médecine Translationnelle de Strasbourg (FMTS), F-67000 Strasbourg, France; 2 Hôpitaux Universitaires de Strasbourg, Anesthésie, Réanimation et Médecine périopératoire, Nouvel Hôpital Civil, F-67000 Strasbourg, France

**Keywords:** sepsis, septic shock, P2Y12 receptor, clopidogrel treatment, antiplatelet drug

## Abstract

**Introduction** Platelets are increasingly appreciated as key effectors during sepsis, raising the question of the usefulness of antiplatelet drugs to treat patients with sepsis.

**Objective** Evaluate the potential contribution of the platelet P2Y _12_ receptor in the pathogenesis of polymicrobial-induced sepsis and septic shock in mice.

**Methods** The effects of P2Y _12_ inhibition using clopidogrel treatment and of platelet-specific deletion of the P2Y _12_ receptor in mice were examined in two severity grades of cecal ligation and puncture (CLP) leading to mild sepsis or septic shock.

**Results** Twenty hours after induction of the high grade CLP, clopidogrel- and vehicle-treated mice displayed a similar 30% decrease in mean arterial blood pressure (MAP) characteristic of shock. Septic shock-induced thrombocytopenia was not modified by clopidogrel treatment. Plasma concentrations of inflammatory cytokines and myeloperoxidase (MPO) were similarly increased in clopidogrel- and vehicle-treated mice, indicating comparable increase in systemic inflammation. Thrombin-antithrombin (TAT) complexes and the extent of organ damage were also similar. In mild-grade CLP, clopidogrel- and vehicle-treated mice did not display a significant decrease in MAP, while thrombocytopenia and plasma concentrations of TNFα, IL6, IL10, MPO, TAT and organ damage reached similar levels in both groups, although lower than those reached in the high grade CLP. Similarly, mice with platelet-specific deletion of the P2Y _12_ receptor were not protected from CLP-induced sepsis or septic shock.

**Conclusion** The platelet P2Y _12_ receptor does not contribute to the pathogenesis of sepsis or septic shock in mice, suggesting that P2Y _12_ receptor antagonists may not be beneficial in patients with sepsis or septic shock.

## Introduction

Sepsis is a life-threatening organ dysfunction caused by an inappropriate host response to an infection. The inflammatory response is dysregulated and leads to a pro-inflammatory status resulting in organ dysfunction and reaching an in-hospital mortality greater than 10%. Septic shock is a subset of sepsis consisting in cellular, metabolic and circulatory abnormalities, leading to a 40% mortality. 1 The inflammatory response is linked to coagulation activation, resulting in bacterial containment, but also in thrombotic microangiopathy through uncontrolled thrombin and fibrin generation, which may evolve toward disseminated intravascular coagulation (DIC). So far, the mainstay of treatment of septic shock relies on the early initiation of a broad-spectrum antibiotic, associated with life-supporting care. Several therapies targeting the inflammatory cascade or the coagulation disorders have been assessed but none of them demonstrated any improvement in the outcome. 2

Thrombocytopenia is frequently encountered in sepsis and septic shock, with an incidence from 15 to 58%. 3 Early thrombocytopenia negatively impacts patient prognosis and may therefore stand as an early predictor of death. 4 However, the association between thrombocytopenia and clinical outcome does not establish causality. In addition, it is not yet clear whether thrombocytopenia serves as a marker for disease severity or represents a direct contribution of platelets to disease progression. An additional degree of complexity is related to the paradox of platelets being potentially both deleterious and beneficial during sepsis course.

Platelets can have a beneficial role in host defense against pathogens by limiting both bacterial growth and dissemination through mechanisms including platelet interaction with pathogens, internalization and destruction or the release of antimicrobial mediators. 5 6 7 In addition, platelets aid in maintaining vascular integrity in the context of a strong pro-inflammatory environment. 8 9 Experimental thrombocytopenia in mice has been shown to result in uncontrolled growth and dissemination of bacteria, increased systemic inflammation, tissue damage and mortality during sepsis as well as hemorrhage at site of primary infection. 8 10 On the other hand, platelets contribute to immune cell recruitment and hyper-inflammation by interacting with leukocytes and vascular endothelial cells, both directly by contact-dependent mechanisms and indirectly through immune mediator secretion 6 11 12 13 and pro-inflammatory vesicle release. 14 Platelets also contribute to multiple organ failure via their thrombotic potential resulting in thrombotic microangiopathy and DIC. 15 16

Inhibition of platelet hemostatic functions may therefore be considered of potential benefit for attenuating tissue injury and improving outcomes in sepsis and septic shock. Antiplatelet drugs such as acetylsalicylic acid (ASA) and P2Y _12_ receptor targeting drugs, including the thienopyridine compounds clopidogrel and prasugrel and direct antagonists such as ticagrelor, are the cornerstone of the treatment and secondary prevention of arterial thrombosis. 17 18 However, clinical studies related to antiplatelet therapy of sepsis generated discordant results with several studies showing a survival benefit in septic patients receiving ASA 19 20 21 or antiplatelet agents (ASA and others), 22 23 while others showed no better outcome after ASA 24 25 or antiplatelet agents. 26 27 In addition, there are only few reports on the effects of antiplatelet drugs in animal models of sepsis. 8 13 GPIIbIIIa blockade with eptifibatide had only a modest effect, if any, on mortality when administered 12 hour after the induction of CLP in mice. 28 The potential benefit of ASA in murine models of sepsis has been reported, 29 30 31 though it remains unclear whether its beneficial effect relies solely on its anti-platelet activity or results from its platelet-independent anti-inflammatory and anti-oxidant properties. Concerning clopidogrel, conflicting results have been reported in various animal models of sepsis with either no protective role 32 33 or a reduced sepsis-induced inflammation. 34

We therefore sought to evaluate the potential contribution of the platelet P2Y _12_ receptor in the pathogenesis of polymicrobial-induced sepsis and septic shock in mice. We examined the effects of P2Y _12_ inhibition using clopidogrel treatment. In addition, mice with platelet-specific deletion of the P2Y _12_ receptor were generated to investigate potential off-targets effects of clopidogrel or a role for the P2Y _12_ receptor possibly expressed in other cells of the immune system or in vascular smooth muscle cells. 35 Since the pathophysiology of sepsis can be greatly affected by the severity grade of disease, we used two severity grades of cecal ligation and puncture (CLP) leading to mild sepsis or septic shock. In each condition, the hallmark responses of mild-sepsis or septic shock were evaluated by measuring systemic inflammation, arterial blood pressure, coagulation and organ damage.

## Materials and Methods

### Materials

Xylazine (Rompun ^®^ ) and ketamine (Imalgene 1,000 ^®^ ) were from Bayer (Leverkusen, Germany) and Merial (Lyon, France), respectively. Isoflurane (Vetflurane ^®^ ) was from Virbac (Cahors, France) and recombinant hirudin from Transgene (Illkirch-Graffenstaden, France). Clopidogrel was provided by Sanofi-Aventis (Paris, France).

### Mice

Experiments were performed using 8 to 10 week-old male C57BL6/J mice purchased from Charles River Laboratories (l'Arbresle, France). To generate mice with the deletion of P2Y _12_ receptor exclusively in the megakaryocytic lineage (PF4-P2Y _12_^−/−^ ), the mice with floxed alleles (P2Y12 ^flox/flox^ mice) were crossed with mice carrying the Cre recombinase under the control of the platelet factor 4 (PF4). P2Y12 ^flox/flox^ (WT) littermates were used as control. Genotyping was performed on mouse tail DNA using a polymerase chain reaction (PCR) amplification method. All mice were housed in the animal facilities of the EFS-Grand Est (agreement number F67 482–10). Ethical approval for the animal experiments was received from the French Ministry of Research in accordance with the guidelines of the European Union and the Guide for the Care and Use of Laboratory Animals.

### Cecal Ligation and Puncture Model

Cecal ligation and puncture (CLP) was performed essentially as described previously. 36 Briefly, C57BL6/J male mice were anesthetized with isoflurane (Vetflurane ^®^ ). Under aseptic conditions, a 1.5 cm midline laparotomy was performed to allow exposure of the cecum with adjoining intestine. Two different severity grades of sepsis were induced through modulation of the position of cecal ligation and the number of puncture. High-grade form of CLP was obtained by ligation of 100% of cecal length just below the ileocecal valve with 3.0 silk and the ligated part of the cecum was punctured twice with a 21-gauge fine needle. Mid-grade form of CLP was obtained by ligation of 50% of cecal length and one single puncture with a 21-Gauge fine needle. A small amount of stool was extruded to ensure patency. The punctured cecum was then placed back into the abdomen; peritoneal wall was closed with continuous sutures using 3–0 silk and skin was closed with interrupted sutures using 3–0 silk. All mice received subcutaneous 1 mL isotonic saline after abdominal closure and 0.1 mg/kg buprenorphine. Mice subjected to sham laparotomy underwent the same procedure but without ligation and puncture of the cecum. After the procedure, mice were placed into a warm enclosure for a 1 hour recovery period then placed into their cages with free access to water and chow. Twenty hours after surgery, mice were anesthetized with isoflurane for measurement of arterial pressure and collecting blood and tissues.

### Clopidogrel Treatment

Clopidogrel (50 mg/kg in 5% arabic gum in water) or vehicle (5% arabic gum in water) was administered to mice *per os* (p.o.) 16 and again 2 hours prior surgery, to irreversibly inhibit the P2Y _12_ receptor. 35

### Mean Arterial Blood Pressure

The left carotid artery of anesthetized mice with isoflurane was exposed and used to measure mean arterial pressure (MAP) using a polyurethane catheter (PUFC-C20–10; Instech Solomon), secured with silk and attached to the data acquisition system (EMKA Instruments).

### Hemostatic Parameters and Enzyme Immunoassays

Blood was drawn into a plastic syringe containing citrate (3.8%) anticoagulant from intracardiac puncture of mice anesthetized with isoflurane. Blood was centrifuged at 3,500 g for 5 minute at 4°C. The supernatant was centrifuged again at 12,000 g for 5 minute to prepare platelet poor plasma, which was stored at -80°C until use. Thrombin-antithrombin complexes (TAT) in plasma were quantified by ELISA (Enzygnost TAT, Behringwerke AG) using calibration standards of human origin (2 to 60 ng/mL TAT). Levels of tumor necrosis factor-α (TNF-α) and interleukin-10 (IL-10) (R&D Systems), interleukin-6 (IL-6, USCN Life Science), myeloperoxidase (MPO, HyCult Biotechnology), aspartate aminotransferase (ASAT, Cloud-Clone Corp.), lactate dehydrogenase (LDH, Abcam) and blood urea nitrogen (BUN, Sigma Aldrich) in plasma were measured by enzyme immunoassay using murine ELISA kits according to the manufacturers' instructions.

### Blood Cell Counts

Platelet and white blood cell (WBC) counts were determined in whole blood collected into EDTA (6 mmol/L) by severing the tail end of anesthetized mice, using a Scil Vet ABC automatic cell counter (Scil Animal Care Company) set to murine parameters.

### Statistical Analyses

Statistical analyses were performed with GraphPad software (Prism 5.02). Data are reported as the mean ± SEM. Shapiro-Wilk test was used for testing normality of all samples. Since datasets had a nonparametric distribution, they were analyzed by Kruskal-Wallis 1-way ANOVA followed by Dunn's post-hoc test. A p value of < 0.05 was considered to be statistically significant.

## Results

### Clopidogrel Treatment does not Impact Adverse Systemic Hypotension during Septic Shock

**Fig. 1:**
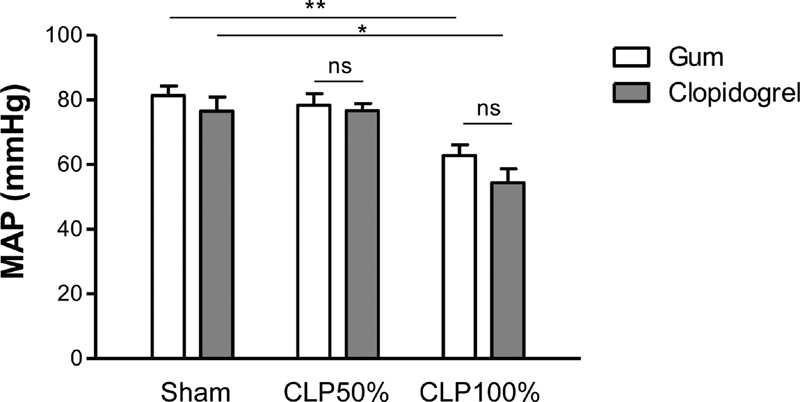
Clopidogrel treatment does not impact adverse systemic hypotension during septic shock. Mean arterial pressure (MAP) in clopidogrel- or vehicle-treated mice, 20 hours after mid- or high-grade form of CLP as compared with sham-surgery. Results are presented as the mean ± SEM ( *n* = 5–7, **p < 0.01, *p < 0.05, ns p > 0.05).

We first evaluated the potential of P2Y _12_ receptor antagonism with clopidogrel treatment in the pathogenesis of polymicrobial-induced sepsis. Clopidogrel (50 mg/kg) was administered to mice p.o. 16 and again 2 hours prior surgery, to fully and irreversibly inhibit the platelet P2Y _12_ receptor for at least 20 hours ( Supplementary Fig. 1 ). Since the pathophysiology of sepsis varies with the severity of disease, we evaluated two defined severity grades (mid or high) of CLP surgery inducing sepsis or septic shock, respectively. Septic shock is characterized by systemic hypotension resulting in tissue hypoxia and organ failure due to hypoperfusion. Indeed, twenty hours after surgery, only high-grade CLP led to a significant 30% decrease in mean arterial pressure (MAP) as compared with sham-operated mice (**p < 0.01) ( Fig. 1 ). This drop in MAP was similar between vehicle- and clopidogrel-treated mice ( Fig. 1 ), indicating that clopidogrel treatment had no beneficial effect on adverse hemodynamic alterations associated with the progression of septic shock.

### Clopidogrel Treatment does not Modify Thrombocytopenia during Sepsis and Septic Shock

**Fig. 2:**
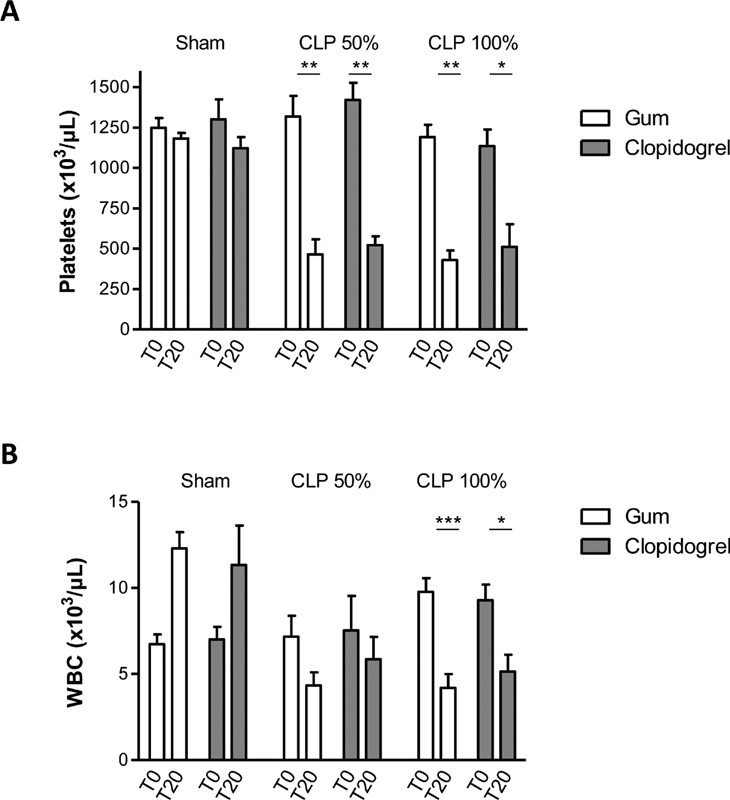
Clopidogrel treatment does not modify thrombocytopenia during sepsis and septic shock. Circulating platelet counts ((A)) and WBC counts ((B)) in clopidogrel- or vehicle-treated mice, before (T0) and 20 hours (T20) after mid- or high-grade form of CLP as compared with sham-surgery. Results are presented as the mean ± SEM ( *n* = 6–7, ***p< 0.001, ** *p* < 0.01, *p < 0.05, ns p > 0.05).

Twenty hours after mid- or high-grade form of CLP surgery, vehicle-treated mice displayed similarly and profoundly decreased platelet counts as compared with sham-operated mice ( Fig. 2A ). These decreased platelet counts were not affected by clopidogrel treatment after mid- or high-grade CLP surgery. WBC counts decreased similarly in clopidogrel- and vehicle-treated mice after high-grade CLP surgery ( Fig. 2B ). These results indicated that clopidogrel treatment did not modify the thrombocytopenia and leukopenia occurring during sepsis or septic shock.

### Clopidogrel Treatment does not Modify Inflammatory Cytokines during Sepsis and Septic Shock

**Fig. 3:**
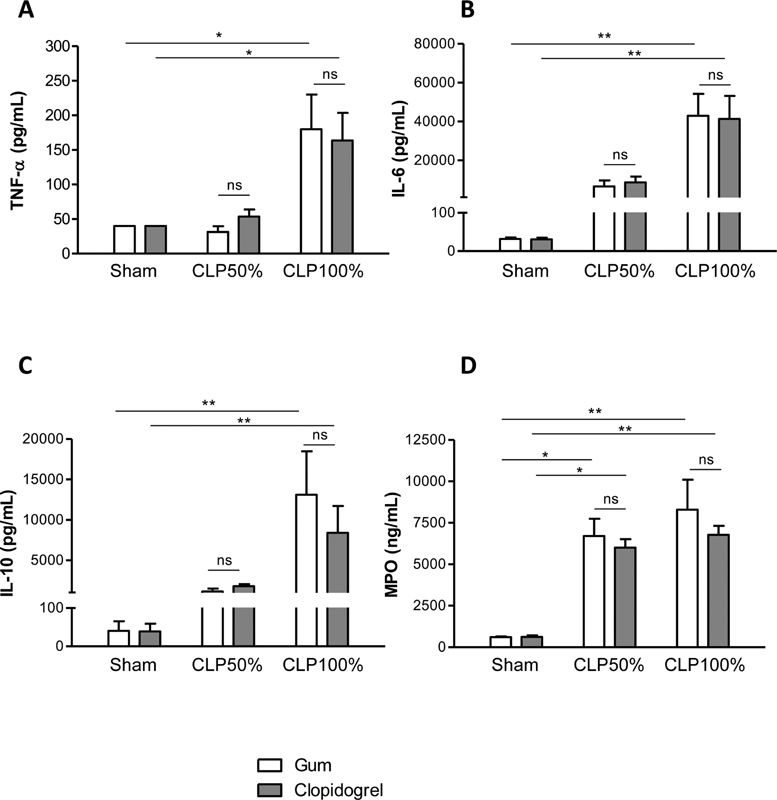
Clopidogrel treatment does not modify the inflammatory response during sepsis and septic shock. Plasma levels of the pro-inflammatory cytokines TNF-α ((A)), IL-6 ((B)), IL-10 ((C)), and of myeloperoxidase (MPO) ((D)) in clopidogrel- or vehicle-treated mice, 20 hours after mid- or high-grade form of CLP as compared with sham-surgery. Results are presented as the mean ± SEM ( *n* = 6–7, **p < 0.01, *p < 0.05, ns p > 0.05).

High levels of circulating pro-inflammatory cytokines play a fundamental role in the development of sepsis, as they activate the innate immune system and are involved in excessive neutrophil accumulation into tissues. To obtain insight in the systemic inflammatory response, we measured plasma levels of the main pro-inflammatory cytokines TNF-α and IL-6 and of the anti-inflammatory cytokine IL-10, 20 hours after surgery. Plasma levels of the two pro-inflammatory cytokines increased progressively with the severity of sepsis, reaching similar levels in clopidogrel- and vehicle-treated mice ( Fig. 3A,B ). Plasma levels of the anti-inflammatory cytokine IL-10 increased also progressively with the severity of sepsis, reaching similar levels in clopidogrel- and vehicle-treated mice ( Fig. 3C ). These results indicated that clopidogrel treatment had no significant impact on the inflammatory status during sepsis and septic shock in mice.

We also evaluated systemic neutrophil degranulation assessed by measuring plasma myeloperoxidase (MPO) concentration. After mid- or high-grade form of CLP surgery, vehicle-treated mice displayed a significant increase in plasma MPO levels compared with sham-operated mice ( Fig. 3D ). Clopidogrel treatment led to similar increase in MPO concentration ( Fig. 3D ), indicating that clopidogrel treatment did not modify systemic neutrophil activation during sepsis and septic shock.

### Clopidogrel Treatment does not Modify Coagulopathy during Sepsis and Septic Shock

**Fig. 4:**
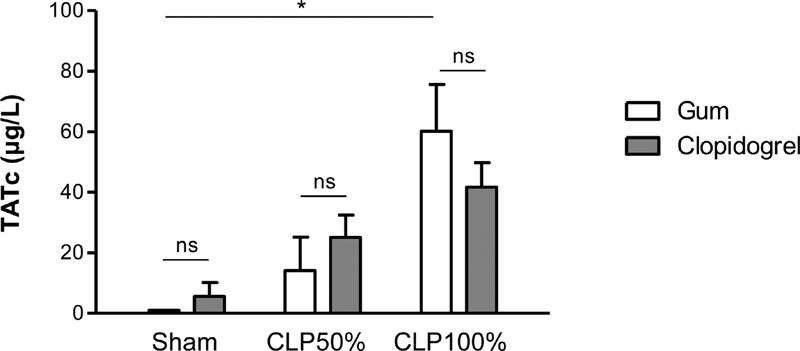
Clopidogrel treatment does not modify coagulopathy during sepsis and septic shock. Plasma levels of thrombin-antithrombin complex (TATc) levels in clopidogrel- or vehicle-treated mice, 20 hours after mid- or high-grade form of CLP as compared with sham-surgery. Results are presented as the mean ± SEM ( *n* = 6–7, *p < 0.05, ns p > 0.05).

To obtain insight into the systemic coagulation activation during sepsis, we measured thrombin-antithrombin complex (TATc) levels in plasma of the different groups of mice. Twenty hours after surgery, all groups of CLP-operated mice displayed an increase in TATc levels as compared with sham-operated mice and there was no difference between clopidogrel- and vehicle-treated mice ( Fig. 4 ). These results indicated that clopidogrel had no impact on coagulopathy during sepsis.

### Clopidogrel Treatment does not Protect Mice from Sepsis-induced Organ Damage

**Fig. 5:**
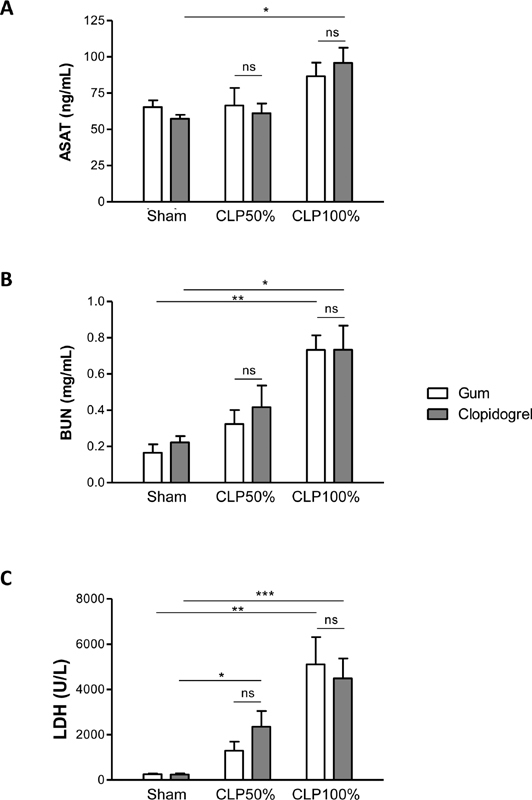
Clopidogrel treatment does not protect mice from sepsis-induced organ damage. Plasma levels of aspartate aminotransferase (ASAT) ((A)), blood urea-nitrogen (BUN) ((B)) or lactate dehydrogenase (LDH) ((C)), in clopidogrel- or vehicle-treated mice, 20 hours after mid- or high-grade form of CLP as compared with sham-surgery. Results are presented as the mean ± SEM ( *n* = 6–7, ***p< 0.001, **p < 0.01, *p < 0.05, ns p > 0.05).

We evaluated organ dysfunction in septic mice by measuring plasma levels of aspartate aminotransferase (ASAT) as an indicator of liver damage, blood urea-nitrogen (BUN) as an indicator of renal dysfunction and lactate dehydrogenase (LDH) as a marker for cell injury. Plasma levels of the three markers increased progressively with the severity of sepsis in vehicle-treated mice compared with sham-operated mice ( Fig. 5 ). The levels of the three markers were not modified in clopidogrel-treated mice in either mid- or high-grade CLP ( Fig. 5 ), indicating that clopidogrel treatment did not protect mice from sepsis or septic shock-induced organ damage.

### Platelet-specific P2Y _12_ Receptor Deficiency does not Protect Mice from Sepsis or Septic Shock

**Fig. 6:**
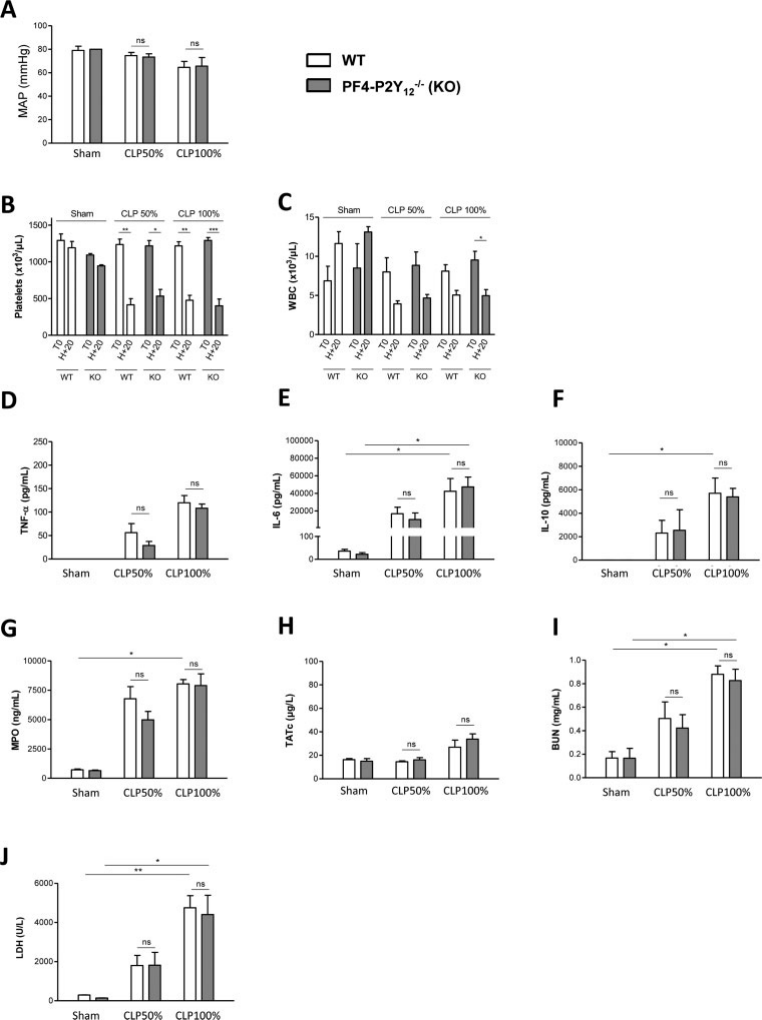
Platelet-specific P2Y _12_ receptor deficiency does not protect mice from sepsis or septic shock. Mean arterial pressure (MAP) ((A)), circulating platelet counts ((B)), WBC counts ((C)), plasma levels of TNF-α ((D)), IL-6 ((E)), IL-10 ((F)), myeloperoxidase (MPO) ((G)), thrombin-antithrombin complex (TATc) ((H)), blood urea-nitrogen (BUN) ((I)), lactate dehydrogenase (LDH) ((J)), in WT or PF4-P2Y _12_^−/−^ mice (KO), 20 hours after mid- or high-grade form of CLP as compared with sham-surgery. Results are presented as the mean ± SEM ( *n* = 6–9, ***p< 0.001, **p < 0.01, *p < 0.05, ns p > 0.05).

We next evaluated mice with platelet-specific deletion of the P2Y _12_ receptor. PF4-P2Y _12_^−/−^ mice displayed normal blood cell counts but a profoundly reduced aggregation in response to various agonists and a severely prolonged bleeding time, similar to those observed when WT mice were treated with high dose (50 mg/kg) of clopidogrel ( Supplementary Fig. 2 ). WT and PF4-P2Y _12_^−/−^ mice were subjected to either mid- or high-grade CLP surgery and the pathology of sepsis was evaluated 20 hours later. After high- but not mid-grade CLP surgery, the MAP of WT and PF4-P2Y _12_^−/−^ mice fell similarly by 20%, indicating systemic hypotension characteristic of shock ( Fig. 6A ). Thrombocytopenia and leukopenia occurred to the same extent after mid- or high-grade CLP surgery in WT and PF4-P2Y _12_^−/−^ mice ( Fig. 6B,C ). Plasma concentrations of the pro-inflammatory cytokines TNF-α and IL-6, of the anti-inflammatory cytokine IL-10, and of myeloperoxidase (MPO), increased progressively with sepsis gravity with no difference between WT and PF4-P2Y _12_^−/−^ mice ( Fig. 6D,E,F, G ). TATc increased only in the case of high grade CLP surgery and to similar extent in WT and PF4-P2Y _12_^−/−^ mice ( Fig. 6H ). The markers of tissue injury (BUN, LDH), which progressively increased with sepsis gravity, were found similar between WT and PF4-P2Y _12_^−/−^ mice ( Fig. 6I,J ). These results indicated that specific platelet P2Y _12_ deletion had no impact on inflammation, coagulopathy and organ injury during CLP-induced sepsis or septic shock.

## Discussion

Platelets are increasingly appreciated as key effectors during sepsis, raising the question of the usefulness of antiplatelet drugs to treat patients with sepsis. Here, we used mice with platelet-specific deletion of the P2Y _12_ receptor and clopidogrel treatment to evaluate the role of the P2Y _12_ receptor in the host response during polymicrobial abdominal sepsis or septic shock and associated organ failure. P2Y _12_ receptor deficiency and receptor antagonism provided similar results indicating that the P2Y _12_ receptor does not contribute to the pathogenesis of sepsis or septic shock in mice, suggesting that antiplatelet therapy with P2Y _12_ receptor antagonists may not be beneficial in septic patients.

Platelet P2Y _12_ receptor signaling is known to influence immune responses. Clopidogrel, the most widely used thienopyridine antiplatelet drug, selectively binds to the P2Y _12_ receptor to inhibit platelet aggregation and block the cascading amplification effect of platelet activation. 17 35 Clopidogrel may attenuate platelet expression of inflammatory and immune markers and the release of inflammatory cytokines. 37 38 39 40 Use of clopidogrel has been coupled with reductions in C-reactive protein (CRP) levels and decreased expression of CD40, CD40L, P-selectin and leukocyte-platelet aggregates in a variety of disease states, including cardiovascular disease, cerebrovascular disease, diabetes, and renal transplantation, 38 39 41 42 suggesting that it could also potentially affect sepsis severity.

Thrombocytopaenia below 50G/L is a strong negative prognostic marker in patients with sepsis and is thought to result from platelet activation and consumption. 4 In mouse models of sepsis, less severe platelet drop is associated with better prognostic, probably due in part to the role of platelets in antibacterial defense. 8 Our results indicating that P2Y _12_ deficiency or receptor antagonism did not modify thrombocytopenia during polymicrobial abdominal sepsis or septic shock rendered unlikely a beneficial impact on host defense. Platelets have been also implicated in inflammatory responses, including leukocyte recruitment and proinflammatory cytokine production during sepsis. 7 10 43 Neutrophil degranulation (as measured by MPO) and plasma cytokine levels were similar between WT and P2Y _12_ deficiency or receptor antagonism. These data suggest that the platelet P2Y _12_ receptor does not contribute to inflammatory responses during CLP-induced sepsis or septic shock in mice. Besides inflammatory reactions, platelets have been implicated in procoagulant reactions, and thereby in the development of sepsis complications such as microvascular dysfunction, disseminated intravascular coagulation, and multiple organ failure. 7 10 43 Our data indicated that platelet P2Y _12_ receptor does not contribute to coagulopathy and to various organ damage during CLP-induced septic shock in mice. This lack of protective role of clopidogrel in mouse polymicrobial abdominal sepsis has also been observed in mouse *S. pneumoniae-* or *K. pneumoniae* -induced pneumosepsis, 32 33 suggesting that the absence of contribution of the P2Y _12_ receptor in sepsis applies to different territories and in response to various bacterial pathogens.

Our findings contrast with a previous study reporting that clopidogrel treatment or P2Y _12_ gene knockdown reduced inflammation in a mouse model of CLP. 34 However, in the absence of measurement of plasma markers of organ damage, it may be premature to conclude from this study on a contributing role of the P2Y _12_ receptor in sepsis. In addition, in this study, 34 septic mice showed a significant increase in WBC counts as compared with the sham-operated mice, which contrasts with the well-established severe leukopenia observed otherwise in the model of CLP-induced sepsis in mice, due to exhaustion of leukocytes in their effort to eliminate the pathogenic bacteria. In addition, septic mice displayed unaltered platelet counts, 34 further contrasting with the severe thrombocytopenia normally observed in CLP mice and septic patients, overall questioning the relevance of their CLP model with respect to the human pathology. Another difference is that Liverani et al used whole-body P2Y _12_ receptor-deficient mice, while we used mice with platelet specific deletion of the P2Y _12_ receptor. Whether a potential contribution of the P2Y _12_ receptor expressed on other cell types could explain the divergent results is presently unknown. The study by Rahman et al 44 showed that pretreatment with ticagrelor, a reversibly-binding platelet P2Y _12_ receptor inhibitor, reduced pulmonary neutrophil recruitment and lung injury in CLP-induced sepsis in mice. Ticagrelor was administered at a dose of 100 mg/kg, resulting in complete inhibition of ADP-induced platelet aggregation, similar to that induced by clopidogrel (50 mg/kg). However, unlike clopidogrel, ticagrelor inhibits cell reuptake of adenosine, 45 which not only has an inhibitory effect on platelet responses but also exerts several different anti-inflammatory effects 46 that may contribute to its protective effect.

In a different model of peritoneal contamination and infection (PCI) induced by intraperitoneal injection of human feces in mice, 47 clopidogrel pretreatment attenuated the decrease in hemostatic potential (illustrated by a decrease in ROTEM clot formation rate (CFR)) observed 6 hours after induction of PCI. The PCI model is very different from the CLP model, as the gut microbiota of mice and humans is not the same and may influence the characteristics of the inflammatory response in this model. In addition, the decrease in hemostatic potential appears in contradiction with the data obtained in clinical studies, showing that sepsis, at least in its early state, is associated with hypercoagulability. 16 Moreover, since the mice were sacrificed 6 hours after PCI, no conclusions can be drawn concerning the impact of clopidogrel at a later time point, especially since effects on pro- or anti-inflammatory biomarkers have not been reported. In another model of LPS-induced inflammation in rat, pro-inflammatory cytokines (IL-6 and TNF-α) as well as lung and liver damage were attenuated upon treatment with clopidogrel. 48 However, LPS infusion model differs from the CLP model in that it does not involve bacteremia, which represents a bias. The innate immune system may have a detrimental effect in the case of the injection of LPS, while it is also beneficial in fighting bacterial dissemination throughout the whole organism. Thus, extrapolating the results of this study to the far more complex situation of sepsis would be too speculative.

Our findings indicating a lack of any beneficial impact of clopidogrel treatment on the pathogenesis of sepsis and septic shock in mice, question whether clopidogrel treatment might be beneficial in septic patients. Numerous clinical trials have generated variable results with either beneficial effect of antiplatelet drugs 19 20 21 22 23 49 or no effect 24 25 26 27 on sepsis severity or outcome. However, most clinical studies were retrospective, of small size and/or included patient groups that were not matched for potential confounding factors, such as comorbidity and other chronic medication. In addition, the potential specific benefit of clopidogrel could not be distinguished because many patients with prescriptions for clopidogrel had one or more indications for lifetime ASA or other medications for prevention of cardiovascular disease. The sole clinical study reporting the effects of clopidogrel on sepsis outcome showed that the use of clopidogrel (75 mg/day received for at least two days during the ICU stay) was not associated with a decrease in mortality. 21 In addition, the post-hoc analysis of the PLATO clinical trial showed no improvement regarding sepsis-related severity and mortality in patients receiving clopidogrel compared with patients not exposed to clopidogrel. 50 Of note, a prospectively assembled cohort of 972 patients admitted to ICU for sepsis, has not confirmed the results of several retrospective analysis, showing no improvement in mortality in-patient on a pre-existing antiplatelet regimen. 27

CLP is considered one of the most clinically relevant models of sepsis. 51 CLP involves a combination of three insults: tissue trauma due to laparotomy, necrosis caused by ligation of the cecum, and infection due to the leakage of peritoneal microbial flora into the peritoneum. One advantage of CLP is that the pathogens originate from within the host, therefore mimicking traumatic injury leading to peritonitis in humans. It reproduces the dynamic changes in cardiovascular function as well as the progressive release of pro-inflammatory mediators seen in humans with sepsis. Nevertheless, this study has some limitations. The mice were sacrificed at 20 hours post-CLP, precluding conclusions about changes that might occur before and after this time point. In addition, mice did not receive the intensive care interventions (antibiotics, inotropes/vasopressors, fluid filling and mechanical ventilation) that are standard of care in human patients. Only male mice were used that were young, healthy, and had not experienced previous insults. This reduces confounding variables in the CLP model but limits the possibility to generalize our data to a human population with sepsis.

Overall, our findings indicate that the platelet P2Y _12_ receptor does not contribute to the pathogenesis of sepsis or septic shock in mice, suggesting that anti-platelet therapy with P2Y _12_ receptor antagonists may not be beneficial in patients with septic shock. Future studies should address how do platelets respond to inflammation and infection and what approaches can be made to preserve host defenses while downregulating unwanted inflammation and host tissue damage.

## Notes

**What is Known about the Topic?**

- The usefulness of antiplatelet drugs to treat patients with sepsis is not established.

**What does this Paper Add?**

- We evaluated the potential contribution of platelet P2Y _12_ receptor to sepsis in mice.
- P2Y _12_ receptor deficiency or P2Y _12_ inhibition with clopidogrel does not protect mice from sepsis.
- Antiplatelet therapy with P2Y _12_ receptor antagonists may not be beneficial in septic patients.
