# Supplementary material for: Platelet P2Y 12 Receptor Deletion or Pharmacological Inhibition does not Protect Mice from Sepsis or Septic Shock
Source: TH Open. 2021 Aug 24;5(3):e343–52. doi: 10.1055/s-0041-1733857 (PMC8384481; doi:10.1055/s-0041-1733857)
Supplement: Supplementary file 1 — Supplementary Material [file 10-1055-s-0041-1733857-s210024.pdf]

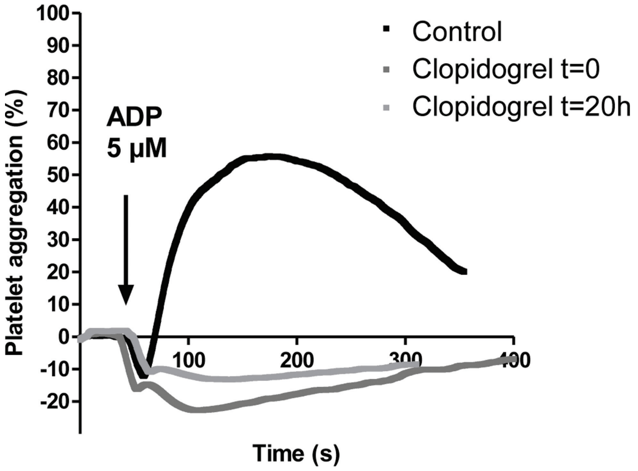

Supplementary Fig. 1 Clopidogrel (50 mg/kg in 5% arabic gum in water) or vehicle (5% arabic gum in water) was administered to mice per os (p. o.) 16 h and again 2 h prior anesthesia and blood collection. Platelet aggregation was measured in cPRP in response to ADP (5 µM) immediately (t=0) or 20 h (t=20 h) later.

 © 2021. The Author(s).

A

	WT	PF4-P2Y$_{12}$$^{-/-}$
White blood cells (x10^3/μL)	7.77 ± 0.97	8.71 ± 0.74
Neutrophils (x10^3/μL)	2.20 ± 0.47	2.07 ± 0.30
Lymphocytes (x10^3/μL)	5.29 ± 0.64	6.32 ± 0.52
Monocytes (x10^3/μL)	0.28 ± 0.04	0.31 ± 0.03
Eosinophils (x10^3/μL)	0.064 ± 0.010	0.067 ± 0.005
Platelets (x10^3/μL)	1245 ± 45	1198 ± 37
Red blood cells (x10^5/mL)	8.13 ± 0.21	8.24 ± 0.20

B

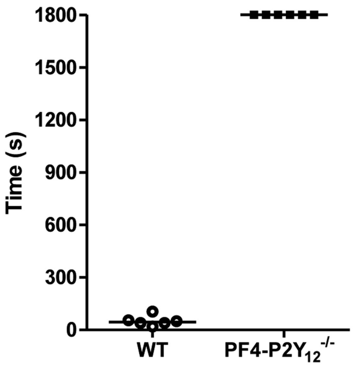

C

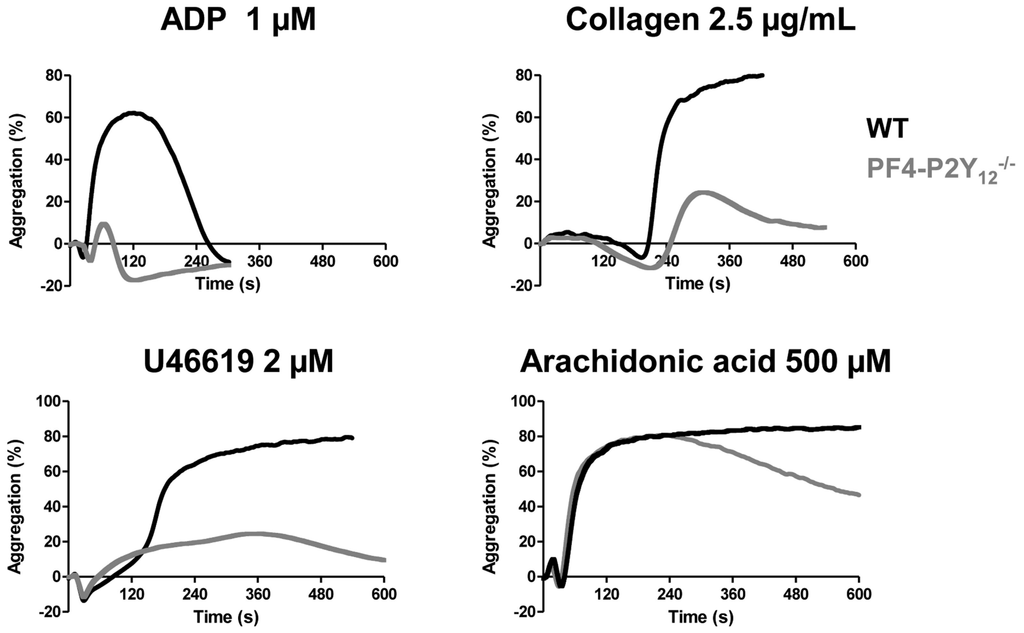

Supplementary Fig. 2. Blood cell counts, tail bleeding times and in vitro platelet aggregation profiles of PF4-P2Y12-/- mice. A. Blood cell counts in WT and PF4-P2Y12-/- mice. Blood cell counts were determined using a Scil Vet ABC automatic cell counter (Scil Animal Care Company) set to murine parameters. Results are presented as the mean ± SEM (n=10). B. Bleeding time measured as the time to the first cessation of bleeding. For PF4-P2Y12-/- mice, bleeding was manually stopped at 1800 s (n=6). C. Platelet aggregation profiles to various agonists in cPRP. Responses to ADP (1 μM), collagen (2.5 μg/mL), U46619 (2 μM) or arachidonic acid (500 μM).

 © 2021. The Author(s).
